# Supplementary material for: Uptake of cervical cancer screening and its determinants in Africa: Umbrella review
Source: PLoS One. 2025 Jul 21;20(7):e0328103. doi: 10.1371/journal.pone.0328103 (PMC12279115; doi:10.1371/journal.pone.0328103)
Supplement: S2 File — (PDF) [file pone.0328103.s002.pdf]

Searching strategies

Google Scholar: ((((((((((Systematic review AND meta-analysis) OR (Systematic reviews)) AND (Uptake) OR (acceptance)) OR (Practice)) OR (utilization)) AND ("Cervical Cancer Screening")) OR (screening)) OR (early cervical cancer diagnosis)) AND (Africa)) OR (Sub-Saharan Africa))

PubMed: ((((((((((Systematic review AND meta-analysis) OR (Systematic reviews)) AND (Uptake) OR (acceptance)) OR (Practice)) OR (utilization)) AND ("Cervical Cancer Screening")) OR (screening)) OR (early cervical cancer diagnosis)) AND (Africa)) OR (Sub-Saharan Africa)) AND (2014/1/1 - 2024/9/20))

Science Direct: "Systematic review AND meta-analysis" AND "Uptake" OR "acceptance" OR utilization OR "Cervical Cancer Screening" AND “Africa” OR “Sub-Saharan Africa”

Hinari: Systematic review AND meta-analysis OR systematic reviews AND uptake OR acceptance OR Practice OR utilization AND "Cervical Cancer Screening" OR screening OR early cervical cancer diagnosis AND Africa OR Sub-Saharan Africa

ScienceDirect: (Systematic review AND meta-analysis) AND (Uptake) OR (utilization) AND ("Cervical Cancer Screening", cervical cancer AND (Africa)) OR (Sub-Saharan Africa)
