# Supplementary material for: Uptake of cervical cancer screening and its determinants in Africa: Umbrella review
Source: PLoS One. 2025 Jul 21;20(7):e0328103. doi: 10.1371/journal.pone.0328103 (PMC12279115; doi:10.1371/journal.pone.0328103)
Supplement: Attachment — Submitted filename: Raised questions and comments for the manuscript.docx [file pone.0328103.s004.docx]

**Raised questions and comments for the manuscript**

**Manuscript ID:** PONE-D-24-50654

**Academic Editor:** Worku Necho Asferie (Assistant Professor of Maternity Health Nursing, Debre Tabor University, Ethiopia)

- Please give appoint to point response the given reviewers comment
- Please submit the revised and track changed manuscript separately

**General Comments**

- The topic is interesting and global issue.
- The manuscript was tried to incorporate necessary components.
- The Manuscript needs major modifications on grammars, spellings, paragraph and sentences writing.
- The PROSPORO registration reference number: CRD42024582302 should include at the end of the abstract part
- Please add line number for your manuscript for easily giving comments.
- Each figure caption should appear directly after the paragraph in which they are first cited.
- Cite references in brackets (for example, “[1]” or “[2-5]” or “[3, 7, 9]”).
- The Supporting Information Captions should be putted at the ends of the reference part.
- The manuscript should include line number

**1. Title**

- Please modify the title as “ Uptake of cercal cancer screening and its determinants in Africa: Umbrella Review”

**2. Abstract**

- It is well addressed but need some modification
- Write “a random-effects model was used to determine the total effect of all studies.” Re-write these sentence as a random-effects model was used to determine the pooled effect estimate.
- Please remove any abbreviation at the abstract part like CCS…
- Please determine the value of I-squared for Heterogeneity of the pooled estimate at the abstract
- Please remove the word “CCS” in the Key word because it is already identified cancer cervical screening in the key word.

**3. Introduction**

- Please make short and concise long sentences from one reference.
- Please show the gaps of other studies which impress you to conduct this umbrella review?
- In general this part needs modification on grammar, chronological sequences of the idea.
- Please show the severity of not uptake this screening on the target population?

**4. Methods and materials**

a. Study protocol and searching strategies

- Please make concise and attractive for readers
- Did you use any filter while searching the eligible studies
- Please identify the details your searching lines at different databases as supplementary file.

b. Inclusion criteria: the second sentence is not correct paraphrased so, make modification for it.

c. Exclusion criteria: Why you excluded the qualitative review? Did you believe that we cannot include them in the umbrella review?

d. Measurements of outcome variable: Your outcome variables are the following. Please state these outcome variables appropriately.

- Uptake of cervical cancer screening:
- Predictors of CCS

e. Quality Assessment

- Make the first sentence as the past tense. i.e. “The quality of the included article will be evaluated ….”

f. Data synthesis and analysis

- Why you select a random-effects model with Der Simonian and Laird’s estimation method to analyze the pooled estimate?
- Which one is used to display the pooled estimate of your study, Odds ratio or log-odds ratio? Please give your justification?
- Please paraphrase the paragraph

**5. Result**

a. Study selection for included studies
 - Please change the heading as **“Literature Searches”**

- **The Fig-1:** which show PRISMA Flow diagram is not correct because the values of **31,050** is not true. Please it again and make correction.
- How many articles did you screened to identify the full text articles? Please describe the numbered tables of all screened articles?

b. Characteristic of included studies

Please remove the word: “Regarding the AMSTAR 2 quality assessment score, the quality score of each SRM study ranges from 15–16”. You can add this in quality assessment result.

c. Factors associated with cervical cancer screening in Africa

- In you title you said “It’s determinants” please make uniform.

6. **Discussion**

- You can’t compare your findings with any meta-analysis that conducted in Africa example kenya, Ghana etc. please looking your discussion. Since your study site is Africa.
- While comparing the findings with the other findings please justify the difference on focusing methodological difference, statistical difference, study unit e.t.c…..
- Therefore you Discussion needs major modification

7. Limitation of this study: Please add the potentials limitation of this pooled estimate of the study.

8. Implication of this study: Please clearly state the implication of conducting this umbrella review.
