# Supplementary material for: Uptake of cervical cancer screening and its determinants in Africa: Umbrella review
Source: PLoS One. 2025 Jul 21;20(7):e0328103. doi: 10.1371/journal.pone.0328103 (PMC12279115; doi:10.1371/journal.pone.0328103)
Supplement: Attachment — Submitted filename: Response to Reviewers.docx [file pone.0328103.s006.docx]

**January 30, 2025**

**Uptake of cervical cancer screening and its determinants in Africa: Umbrella review**

**Manuscript Number:**PONE-D-24-50654

First of all, the authors would like to thank the PLOS ONE Academic Editorial Team, the Editor who handle this manuscript, and the reviewers who have been reviewing, giving swift and valuable comments and suggestions to improve the quality of the manuscript. We have thoroughly considered every suggestion provided, and the corresponding revisions have been diligently incorporated into the updated version of the manuscript. Your expertise and guidance have been pivotal in enhancing the overall quality of our manuscript. We have incorporated all minor and major comments and suggestions of Academic Editor and Reviewers with track changes in the revised manuscript and a point-by-point response. We kindly invite to take a look a table below:

| **Reviewer’s comments/reflections** | **Authors’ responses** |
| --- | --- |
| **General comments**   - The topic is interesting and global issue. - The manuscript was tried to incorporate necessary components. - The Manuscript needs major modifications on grammars, spellings, paragraph and sentences writing. - The PROSPORO registration reference number: CRD42024582302 should include at the end of the abstract part - Please add line number for your manuscript for easily giving comments. - Each figure captionshould appear directlyafter the paragraph inwhich they are first cited. - **Cite references in brackets (for example, “[1]” or “[2-5]” or “[3, 7, 9]”).** - The Supporting InformationCaptions should be putted at the ends of the reference part. - The manuscript should include line number | - **Dear Reviewer,** thank you for your   valuable time to review our submission. We  are also grateful for your appreciation and  general comments.   - We carried out an extensive modification regarding grammars, spellings, paragraph and sentences writing throughout the manuscript. - The PROSPORO registration reference numberCRD42024582302 mentioned in the **“Method sectionof abstract”.** - A continuous line number added in the manuscript - The figure captions are placed for each figure - The **parentheses** of reference citations are changed into square brackets or []. |
| **Specific comments of reviewers** |  |
| **1. Title**   - Please modify the title as “Uptake of cercal cancer screening and its determinants in Africa: Umbrella Review” | **Thank you for the comment.**  Accordingly,amendment has been made in  the revised manuscript. |
| **2. Abstract**   - It is well addressed but need some modification - Write “a random-effects model was used to determine the total effect of all studies.” Re-write thesesentences as a random-effects model was used to determine the pooled effect estimate. - Please remove any abbreviation at the abstract part like CCS… - Please determine the value of I-squared for Heterogeneity of the pooled estimate at the abstract - Please remove the word “CCS” in the Key word because it is already identified cancer cervical screening in the key word. | **Dear reviewers, thank you for your**  **comments on this part.**   - As per the comments, revision has been   made on the model we used: **“A random-**  **effects model was used to determine the**  **pooled effect estimates”.**   - The abbreviation **“CCS”** removed - The value of I-squared for the pooled estimate mentioned in the “**Result section of abstract”.** |
| **3. Introduction**   - Please make short and concise long sentences from one reference. - Please show the gaps of other studies which impress you to conduct this umbrella review? - *In general, this part needs modification on grammar, chronological sequences of the idea.* - Please show the severity of not uptake this screening on the target population? | - Thank you for this valuable and scientific comments in the introduction section. - We carried out a major modification in the introduction part, we kindly invite to look at the changes in the tracked and revised manuscript. - We have attempted to show the reason why we conducted this umbrella review in the final paragraph of the introduction part. - To date, there are multiple SRM that have found an inconsistent prevalence of CCSuptake with significant heterogeneity across Africa, ranging from the lowest pooled estimate (5.47%) to the highest (43%). Besides, this umbrella review attempt to combined the pooled estimate of CCS uptake and its determinants among different study populations. For instance: eligible-age group women, reproductive age women, and women living with HIV. We believe that the comprehensive finding of this umbrella review will be crucial for health professionals, researchers, policymakers, and concerned NGOs to implementing evidence-based actions to scale up uptake of CCS. Ultimately, it capable the detection and treatment of precancerous cervical lesions, improves survival rates, reduces morbidity and mortality from cervical cancer. |
| **4. Methods and materials**  a. Study protocol and searching strategies   - Please make concise and attractive for readers - Did you use any filter while searching the eligible studies | - **Thank you for your constructive comments in the**   **method section.**   - We underwent some changes to improve readability of the study protocol and search strategies that we follow during this review. - We used different “**filters”** while searching eligible studies in different searching engines such as publication year, type of articles (systematic reviews and meta-analysis), full access articles, sex (female), human studies, published in English language. |
| - Please identify the details your searching lines at different databases as supplementary file. | - **Thank you!** We have prepared searching strategies for different search engines as **supplementary file.** |
| b. Inclusion criteria: the second sentence is not  correct paraphrased so, make modification for it.  c. Exclusion criteria: Why you excluded the  qualitative review? Did you believe that we cannot  include them in the umbrella review? | - **Thank you! We re-write the inclusion criteria.** - We excluded qualitative reviewsbecause ofthey often do not report relevant quantitative data such as the prevalenceof cervical cancer screening uptake and adjusted odds ratio (AOR) of associated factors. These data are crucial for undergoing quantitative statistical analysis for this umbrella review. |
| d. Measurements of outcome variable: Your  outcome variables are the following. Please state  these outcome variables appropriately.   - Uptake of cervical cancer screening: - Predictors of CCS | - **Thank you for this comment.** We made amendments on the main outcome variables of this umbrellareview. We stated the operational definition for the CCS uptake supported by references. All the changes are included in the revised manuscript. |
| e. Quality Assessment   - Make the first sentence as the past tense. i.e. “The quality of the included article will be evaluated ….” | **We appreciate the reviewers’ insight and apologies**  **for the error.** Accordingly, we have changed the future  tense into past tense in the revised manuscript. |
| **f.Data synthesis and analysis**   - Why you select a random-effects model with Der Simonian and Laird’s estimation method to analyze the pooled estimate? | - **We grateful for the following scientific question.** - A random-effects model using Der Simonian and Laird's estimation method successfully accounts for variation (substantial heterogeneity) in effect sizes among studies, resulting in a more reliable and representative pooled estimate for an umbrella review or meta-analysis. It adjusts standard errors to provide a more accurate representation of the overall effect by taking into account both within-study andbetween-study variances. - DIO: [**https://doi.org/10.1016/j.jspi.2009.09.017**](https://doi.org/10.1016/j.jspi.2009.09.017) |
| - **Which one is used to display the pooled estimate of your study, Odds ratio or log-odds ratio? Please give your justification?** | We have displayed the log-odds ratio. For this  umbrella review, the adjusted odds ratio for each SRM  was extracted in Excel sheet. Then, the log-odds ratio  was calculated rather than the odds ratio itself. This is  because the log-odds ratio enables the use of statistical  procedures including the weighted average of different  study estimates, allowing for the aggregation of data  across studies. |
| **5. Result**  a. Study selection for included studies  - Please change the heading as **“Literature**  **Searches”** | **Thank you!** The comment taken and corrected. |
| - **The Fig-1:** which show PRISMA Flow diagram is not correct because the values of **31,050** is not true. Please it again and make correction. - How many articles did you screened to identify the full text articles? Please describe the numbered tables of all screened articles? | **Dear reviewers, thank you for your comment and**  **suggestion in this section.**We carried out an extensive  revision in the PRISMA flowchart. A comprehensive  searching strategy has been developed for selection of studies. As a result, a total of 722 articles has been retrieved from different searching engines. After removing duplicated studies, screened by titles and abstracts, 57 articles were identified for full text review. We kindly, invite the reviewers to look at the modified **“Fig-1”.** In addition, the numbered table has been prepared for inclusion and exclusion of articles **(Additional file 5).** |
| b. Characteristic of included studies   - Please remove the word: “Regarding the   AMSTAR 2 quality assessment score, the quality  score of each SRM study ranges from 15–16”.  You can add this in quality assessment result.  c. Factors associated with cervical cancer  screening in Africa   - In you title you said “It’s determinants” please make uniform. | - The comment has been taken and modifications has been made in this section. - We appreciate the reviewers’ insight for this comment. We have taken the comment and we try to be consistent throughout the document. |
| 6. **Discussion**   - You can’t compare your findings with any meta-analysis that conducted in Africa example kenya, Ghana etc. please looking your discussion. Since your study site is Africa. - While comparing the findings with the other findings please justify the difference on focusing methodological difference, statistical difference, study unit e.t.c….. - Therefore you Discussion needs major modification | **Dear reviewers, thank you in advance for your**  **valuable comment and suggestion in this part.**  Accordingly, we carried out a substantial  modification to compare and contrast the findings of  this umbrella review. All the changes have been  shown by tracked change and incorporated in the  revised manuscript. |
| 7. Limitation of this study: Please add the potentials limitation of this pooled estimate of the study. | **Thank you again!** We have stated the potential  limitations of this pooled estimate. |
| 8. Implication of this study: Please clearly state the implication of conducting this umbrella review. | Lastly, we would like to forward for your comment to  add the implication of conducting this umbrella review.  So, we have mentioned the implication of this umbrella  review has been explained in the bottom of part of the  discussion. |
| **Reviewer’s comments/reflections** | **Authors’ responses** |
| **Reviewer #1:**  **Summary: This is a well-performed systemic**  **review (umbrella review) of systemic reviews**  **and meta-analyses on the prevalence of**  **cervical cancer screening uptake in Africa and**  **factors associated with screening uptake. The**  **study was comprehensive and a thorough**  **effort was put into understanding the accuracy**  **and validity of the data to make claims for a**  **large region. The methods and data could be**  **presented more clearly and concisely. The**  **manuscript would benefit from a discussion of**  **the implications of these findings and**  **suggestions or recommendations for future**  **interventions or policies to improve cervical**  **cancer screening implementation. Finally, the**  **paper would benefit from a critical read for**  **typographical errors, grammatical edits, and**  **overall flow and clarity, which might limit its**  **impact.** | Dear Reviewer #1, thank you for your thorough review  and valuable insights. We appreciate your expertise in  this area. We sincerely apologize for the grammatical  and typographical errors. We attempt to address all  the minor and major comments and suggestions raised  by the reviewer. All modifications are incorporated in  the revised manuscript. |
