# Supplementary material for: Uptake of cervical cancer screening and its determinants in Africa: Umbrella review
Source: PLoS One. 2025 Jul 21;20(7):e0328103. doi: 10.1371/journal.pone.0328103 (PMC12279115; doi:10.1371/journal.pone.0328103)
Supplement: Attachment — Submitted filename: Response_to_Reviewers_auresp_2.docx [file pone.0328103.s007.docx]

**March 30, 2025**

**Uptake of cervical cancer screening and its determinants in Africa: Umbrella review**

**Manuscript Number:** PONE-D-24-50654

Dear **Worku Necho Asferie** (Assistant Professor, Academic Editor), we are delighted to receive your comments for our submission. We are also grateful for the honored reviewers, their scientific comments, expertise, and guidance have been pivotal in enhancing the overall quality of our paper. We have addressed the raised minor comments of Academic Editor. The changes have highlighted with track changes and included in the revised manuscript. Finally, we kindly invite to take a look a point-by-point response in the table below:

| **Reviewer’s comments/reflections** | **Authors’ responses** |
| --- | --- |
| **Please review your reference list**  **to ensure that it is complete and**  **correct.** | - **Dear Academic Editor and Reviewers,** thank you for your valuable time and constructive comments. We have taken the comments regarding the reference list. Accordingly, we have checked all the references and some modifications have been made in the manuscript. - As per the Editor’s and reviewers’ comment, we try to cite published references in place of unpublished evidences. - In addition, some up to date articles are included - The changes are highlighted in Tracked change and included in the revised manuscript. |
| **Any changes to the reference list**  **should be mentioned in the**  **rebuttal letter that accompanies**  **your revised manuscript.** | - Here are some of the reference lists that we included in the revised manuscript: - Chidebe RC, Osayi A, Torode JS. The Global Fund, Cervical Cancer, and HPV infections: what can low-and middle-income countries do to accelerate progress by 2030? - Organization WH. Global strategy to accelerate the elimination of cervical cancer as a public health problem: World Health Organization; 2020. - Bray F, Laversanne M, Sung H, Ferlay J, Siegel RL, Soerjomataram I, et al. Global cancer statistics 2022: GLOBOCAN estimates of incidence and mortality worldwide for 36 cancers in 185 countries. CA: a cancer journal for clinicians. 2024;74(3):229-63. - Arbyn M, Weiderpass E, Bruni L, de Sanjosé S, Saraiya M, Ferlay J, et al. Estimates of incidence and mortality of cervical cancer in 2018: a worldwide analysis. The Lancet Global Health. 2020;8(2): e191-e203. - De Martel C, Plummer M, Vignat J, Franceschi S. Worldwide burden of cancer attributable to HPV by site, country and HPV type. International journal of cancer. 2017;141(4):664-70. - Wilailak S, Kengsakul M, Kehoe S. Worldwide initiatives to eliminate cervical cancer. International Journal of Gynecology & Obstetrics. 2021;155:102-6. |
| **If you have cited papers that have**  **been retracted, please include the**  **rationale for doing so in the**  **manuscript text, or remove these**  **references and replace them with**  **relevant current references.**  **If you need to cite a retracted**  **article, indicate the article’s**  **retracted status in the References**  **list and also include a citation and**  **full reference for the retraction**  **notice.** | - Thank you! We made a double check and we did not include   retracted articles as a reference.   - However, we have already included a single pre-print study, which is eligible for review. - Title: Omolo B, Tladi P, Gabaitiri L, Makubate B. Cervical cancer screening uptake, associated barriers and facilitators: a systematic review and meta-analysis, 2023.   **Thank you in advance!!** |
